# Tolerance for chemotherapy-induced peripheral neuropathy among women with metastatic breast cancer: a discrete-choice experiment

**DOI:** 10.1007/s10549-025-07715-5

**Published:** 2025-05-13

**Authors:** Rotana M. Radwan, Anne L. R. Schuster, Daniel L. Hertz, Maryam B. Lustberg, Hetal R. Vachhani, Erin Hickey Zacholski, Vanessa B. Sheppard, John F. P. Bridges, Teresa M. Salgado

**Affiliations:** 1 Department of Pharmaceutical Outcomes and Policy, College of Pharmacy, University of Florida, Gainesville, FL USA; 2 Department of Biomedical Informatics, College of Medicine, The Ohio State University Wexner Medical Center, Columbus, OH USA; 3 Department of Clinical Pharmacy, College of Pharmacy, University of Michigan, Ann Arbor, MI USA; 4 Department of Medical Oncology, School of Medicine, Yale University, New Haven, CT USA; 5 Department of Internal Medicine, School of Medicine, Virginia Commonwealth University, Richmond, VA USA; 6 Department of Pharmacotherapy and Outcomes Science, School of Pharmacy, Virginia Commonwealth University, Richmond, VA USA; 7 Department of Social and Behavioral Sciences, School of Public Health, Virginia Commonwealth University, Richmond, VA USA; 8 Massey Comprehensive Cancer Center, Virginia Commonwealth University, Richmond, VA USA

**Keywords:** Patient preference* [MeSH], Discrete-choice experiment, Chemotherapy-induced peripheral neuropathy, Metastatic breast cancer

## Abstract

**Purpose:**

To quantify preferences for chemotherapy-induced peripheral neuropathy (CIPN) risks and survival benefits of continuing neurotoxic chemotherapy and explore differences in preferences by race among women with metastatic breast cancer (mBC).

**Methods:**

Women with mBC and CIPN experience completed a discrete-choice experiment that included 12 choice tasks presenting paired profiles that varied four attributes across three levels each: progression-free survival (PFS) (6, 12, 24 months), neuropathy in hands (mild, moderate, severe), neuropathy in feet (mild, moderate, severe), and neuropathy persistence (short-term, long-term, permanent). Aggregate and exploratory stratified (White versus non-White) conditional logit models were estimated from which patients’ minimum acceptable benefit was calculated using the willingness-to-pay approach.

**Results:**

Women (n = 189) were on average 52.5 years and 47.1% were non-White. Fewer women who were non-White held a bachelor’s degree or higher (p < 0.01) and reported a household income of $85,000 or higher (p = 0.03). In both the aggregate and the stratified analyses, women preferred longer duration of PFS, less severe CIPN in hands and feet, and shorter CIPN duration. In aggregate, respondents were willing to tolerate a one-level increase in neuropathy severity (mild to moderate or moderate to severe) in their hands and feet in exchange for 6.7 and 2.9 months of PFS, respectively. In exchange for 9.3 months of PFS, respondents were willing to tolerate a one-level increase in neuropathy persistence (short-term to long-term or long-term to permanent). Exploratory stratified analysis showed that non-White women had different preferences from White women (p < 0.01), with non-White women requiring more months of PFS benefit to tolerate increases in neuropathy severity and duration compared to White women.

**Conclusion:**

Women with mBC favored longer duration of progression-free survival, less severe CIPN in hands and feet, and shorter CIPN duration. Different preferences by race warrant additional future investigation.

**Supplementary Information:**

The online version contains supplementary material available at 10.1007/s10549-025-07715-5.

## Introduction

Chemotherapy-induced peripheral neuropathy (CIPN) is a condition characterized by damage to the peripheral nerves resulting from the administration of neurotoxic chemotherapy agents, including platinum drugs, vinca alkaloids, and taxanes [1, 2]. Within the context of breast cancer, neurotoxic agents are commonly used for the treatment of metastatic breast cancer (mBC) and contribute to the development of CIPN in over 60% of patients [3–5]. CIPN symptoms range from pain, thermal sensitivity, numbness, tingling, muscle weakness, and cramps [4, 6]. Symptoms typically start with loss of sensation or tingling in the fingers and toes, and spread proximally to affect both lower and upper extremities [4, 6]. As such, the likelihood of individuals experiencing falls and sustaining injuries increases [7]. Simultaneously, engaging in manual tasks that require the use of hands and feet, such as typing, writing, sewing, handling tools, cooking, dressing, and driving may pose challenges [7]. Symptoms can progress to severe, irreversible physical impairment, reducing patients’ quality of life for months to years post-therapy, with more severe cases of CIPN being more likely to become permanent [8–11].

Few options exist for preventing or treating CIPN [12, 13]. The American Society of Clinical Oncology (ASCO) guidelines recommend reducing, delaying, or discontinuing neurotoxic chemotherapy in patients with intolerable CIPN [12, 13]. Early discontinuation of treatment can decrease the relative dose intensity, potentially compromising treatment effectiveness (i.e., progression-free survival) [11, 13, 14]. In clinical practice, the decision to continue or discontinue neurotoxic chemotherapy is primarily based on the patient’s current CIPN severity level, without adequately taking into account other crucial disease and patient factors [11]. Continuing neurotoxic chemotherapy involves weighing the benefit of completing more cycles of chemotherapy to achieve progression-free survival against the risk of functional impairment due to CIPN. This decision requires careful consideration of treatment effectiveness, the patient’s tolerance for CIPN symptoms, and the patient’s preference for benefit-risk tradeoffs [11]. For example, it is essential to assess whether a patient is willing to continue treatment despite potential physical deterioration from CIPN, accept the persistence of CIPN symptoms, and determine how much survival benefit would justify their tolerance for CIPN symptoms [11].

We sought to quantify preferences for CIPN risks and survival benefits of continuing neurotoxic chemotherapy among women with metastatic breast cancer (mBC) and explore differences in preferences by race, since CIPN incidence is higher among self-reported Black patients [15, 16]. Knowledge of patient preferences can help oncology providers understand the relative importance patients place on different outcomes and the tradeoffs that they are willing to make, thereby improving patient-clinician communication and allowing for informed treatment decisions [17].

## Methods

### Study design, sample, and recruitment

A discrete-choice experiment (DCE) was developed to elicit patient preferences. This study followed the framework of the International Society for Pharmacoeconomics and Outcomes Research Conjoint Analysis Task Force checklist for good research practices [18].

Eligible participants were adult women with mBC at any stage of neurotoxic treatment (i.e., completed, ongoing, or discontinued) who had experienced or who were currently experiencing CIPN. This broad eligibility criteria ensured the inclusion of diverse perspectives. Participants had to reside in the United States and be able to speak and read English. The target sample size for the experiment was 200 individuals, which is consistent with the range typically observed in published DCE studies [19] and current best practices [20].

The study utilized a multimodal recruitment strategy, including posting flyers in breast cancer social media support groups, featuring them in newsletters disseminated by breast cancer organizations, and encouraging word-of-mouth referrals among patients. Eligible participants were also identified with the assistance of the Cancer Informatics Core from the Virginia Commonwealth University (VCU) Massey Comprehensive Cancer Center and invited to participate via mailed letters. For security purposes, interested individuals contacted the lead author (RR) to receive a personalized survey link via email. Participants recruited from online channels answered screening questions via email to confirm eligibility (e.g., receptor status, line of treatment) before receiving the personalized survey link. For those identified through VCU Massey Comprehensive Cancer Center who did not respond, three follow-up calls were made, and one reminder email was sent. Due to these varied recruitment methods, it was not possible to accurately determine the total number of study invitations sent or calculate a precise response rate. A convenience sample of three oncology clinicians from the VCU Massey Comprehensive Cancer Center was recruited via email to consult during the instrument development process. While clinicians were offered $25 as compensation, two of the three declined payment. Participants in the study were given a $50 gift card as a token of appreciation for their time. The study protocol was deemed exempt by the VCU Institutional Review Board.

### Instrument development

The development of the DCE instrument followed an iterative, community-based approach, consisting of: (i) evidence synthesis, (ii) expert consultation (n = 3), (iii) patient engagement (n = 7), and (iv) pretest interviews (n = 20) [21, 22]. Attributes and levels were identified through a literature review and presented to oncology clinicians, and women with mBC who had experienced CIPN. Clinicians evaluated clinical accuracy, while patients assessed their relevance to personal experiences. Both groups were encouraged to suggest additional attributes and levels. Feedback was received through emails, telephone calls, or virtual meetings, with regular consultations with a patient preference expert (JFB). All changes were discussed with patients for approval.

Four risk–benefit attributes with three levels each were incorporated in the DCE: progression-free survival (6, 12, 24 months), neuropathy in hands (mild, moderate, severe), neuropathy in feet (mild, moderate, severe), and neuropathy persistence (short-term, long-term, permanent). Duration levels for progression-free survival were hypothetical, but clinically relevant. Severity levels for neuropathy in hands and feet were partially derived from the Common Terminology Criteria for Adverse Events (CTCAE) [23, 24]. Duration levels for neuropathy persistence consisted of ‘‘short-term’’ and ‘‘long-term’’ reflecting common terminology used to describe the duration of chemotherapy side effects. ‘‘Short-term’’ was defined as 6 months, and ‘‘long-term’’ as 1 year. Given that patients’ remaining life expectancy varies, we included ‘‘permanent’’ to describe neuropathy that lasts indefinitely. The inclusion of ‘‘permanent’’ neuropathy was driven by prior research emphasizing the lasting and irreversible effects of CIPN [8, 11, 25].

The DCE was pretested with 20 participants using in-depth ‘‘think aloud’’ exercises to assess comprehension, terminology, willingness to tradeoff, and cognitive ease [26]. This process resulted in minor language modifications and clarification as appropriate. Attributes, levels, and their respective descriptions are presented in Table 1. To familiarize respondents with the attributes and levels, participants were asked to rate the importance of each attribute using a three-point scale before completing the DCE (results not reported in this manuscript).

**Table 1:** Description of attributes and levels used in the discrete-choice experiment

Attribute	Attribute description	Levels	Level description
Progression-free survival (PFS)	The length of time that the treatment is effective in controlling the growth of cancer before the cancer starts to grow again	6	6 months of PFS
		12	12 months of PFS
		24	24 months of PFS
Neuropathy in hands	Chemotherapy treatment can cause tingling, numbness, burning or sharp pain, and muscle weakness starting from the fingers and spreading to the hands, wrists, and even elbows. Symptoms may affect your ability to perform activities of daily living, such as doing or undoing buttons or zips, using a telephone, or holding a cup	Mild	Symptoms do not affect activities of daily living
		Moderate	Symptoms affect activities of daily living, but do not completely prevent individuals from doing these activities
		Severe	Symptoms significantly affect activities of daily living, to the point where individuals may require assistance
Neuropathy in feet	Chemotherapy treatment can cause tingling, numbness, burning or sharp pain, and muscle weakness starting from toes and spreading to the feet, ankles, and even knees. Symptoms may affect your ability to perform activities of daily living such as walking, climbing stairs, and running. Symptoms can also increase your risk of falling because of losing balance	Mild	Symptoms do not affect activities of daily living, nor do they increase your risk of falling
		Moderate	Symptoms affect activities of daily living but do not increase your risk of falling
		Severe	Symptoms affect both activities of daily living and increase your risk of falling. Individuals may require physical therapy or the use of mobility aids such as crutches, walkers, or a wheelchair
Neuropathy persistence	The length of time that your neuropathy symptoms continue even after discontinuing the course of treatment that was causing you neuropathy	Short-term	Neuropathy will last 6 months after treatment discontinuation
		Long-term	Neuropathy will last 12 months after treatment discontinuation
		Permanent	Neuropathy will last forever after treatment discontinuation

Preference elicitation was framed within a clinical vignette, where respondents were asked to picture their oncologist informing them about the risks and benefits of continuing neurotoxic chemotherapy. While continuing neurotoxic chemotherapy might improve their progression-free survival, it could also increase the risk of CIPN severity and persistence. Patients were then presented with two scenarios and asked to choose the scenario under which they would be more willing to continue treatment.

**Fig. 1:**
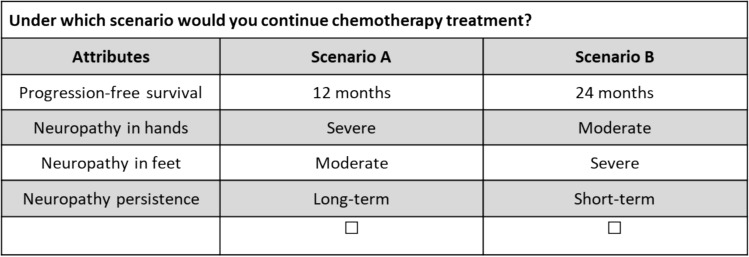
Example of a choice task

Twelve choice tasks were developed using a D-efficient design with zero priors (Fig. 1). Before starting the experiment, participants were provided with an explanation and an example of how to complete a DCE choice task.

### Data collection

The self-administered survey (Appendix A) was distributed via Qualtrics™ (Provo, Utah) over a 12-week period from July to September 2023, and took approximately 30 min to complete. All responses were anonymous, and respondents were required to answer every question and complete all choice tasks, resulting in no missing data. Before beginning the survey, respondents completed screening questions to confirm eligibility, read information about the study procedures, and provided informed consent.

The survey consisted of three sections: clinical history, the DCE, and demographic characteristics. Clinical history included: years since mBC diagnosis, years since CIPN onset, and type of treatment adjustments due to CIPN, such as delays, reductions, or discontinuation. Current experience of CIPN symptoms was assessed using the two-item PRO-CTCAE measure, evaluating the severity of numbness/tingling in the hands and feet (0 = none, 1 = mild, 2 = moderate, 3 = severe, 4 = very severe) and its interference with daily activities (0 = not all, 1 = a little bit, 2 = somewhat, 3 = quite a bit, 4 = very much) over the past seven days [23, 27]. Demographic information included age, race/ethnicity, educational attainment, and income.

### Statistical analysis

Descriptive statistics were computed to characterize the sample, with categorical variables presented as frequencies and percentages, and continuous variables as means and standard deviations (SDs). Bivariate analysis (t-test for continuous and chi-square tests for categorical variables) were performed to compare sample characteristics between women who were White and non-White. Aggregate and stratified DCE models were estimated using a conditional logit model, wherein the dependent variable was the preferred scenario (i.e., scenario A or B) in each choice task, and the independent variables were the levels of each of the attributes in each scenario [28]. Effect coding was used to estimate a coefficient (preference weight) for each attribute level [29]. Statistical differences between the aggregate and the stratified models were examined with the Wald test, with a p < 0.05 indicating statistical difference.

The minimum acceptable benefit (MAB) was calculated using the willingness-to-pay approach by dividing the coefficient of each risk (derived from a continuously coded conditional logit model) by the coefficient for the benefit [30]. The MAB is interpreted as the minimum change in effectiveness that respondents would require (on average) to accept changes to a less desirable level in another attribute. In this study, the MAB estimates the minimum number of months of progression-free survival that would be required to compensate for a one-level increase in the severity (i.e., either from mild to moderate or from moderate to severe) of neuropathy in hands or feet, and the duration (i.e., either from short-term to long-term or from long-term to permanent) of neuropathy persistence [30]. The data were analyzed using Stata 18 (StataCorp LP, College Station, Texas).

## Results

Among the 189 women who completed the survey, 36.0% were recruited through VCU Massey Comprehensive Cancer Center and 64.0% via other channels, including electronic newsletters distributed by breast cancer organizations, and private social media support groups dedicated to breast cancer or CIPN. Participants were on average 52.5 (SD = 12.65) years and about half were non-White (47.1%). Fewer women who were non-White held a bachelor’s degree or higher (p < 0.01) and reported a household income of $85,000 or higher (p = 0.03) (Table 2).

**Table 2:** Participant baseline characteristics

Characteristics	Full sample (n = 189)	White (n = 100)	Non-White (n = 89)	p-value^b^
Age, mean (SD)	52.5 (12.7)	53.0 (12.2)	52.0 (13.2)	0.61
Bachelor’s degree or higher, n (%)	122 (64.6)	74 (74.0)	48 (53.9)	< 0.01
Income greater than $85,000, n (%)	91 (48.2)	56 (56.0%)	35 (39.3%)	0.03
Years since diagnosis, n (%)				
Less than 1 year	21 (11.1)	14 (14.0)	7 (7.9)	0.25
Between 1 and 5 years	113 (59.8)	52 (52.0)	61 (68.5)	0.03
More than 5 years	55 (29.1)	34 (34.0)	21 (23.6)	0.15
Years experiencing CIPN, n (%)				
Less than 1 year	44 (24.3)	22 (22.0)	22 (27.0)	0.60
Between 1 and 5 years	96 (53.0)	48 (49.0)	48 (58.0)	0.30
More than 5 years	41 (22.7)	28 (29.0)	13 (16.0)	0.05
^a^Current CIPN severity, mean (SD)				
Numbness/tingling in hands and feet	1.9 (0.9)	1.9 (0.8)	2.0 (1.0)	0.35
Interferes with usual daily activities	1.8 (1.2)	1.6 (1.1)	2.0 (1.2)	0.03
Adjusted chemotherapy due to CIPN, n (%)	78 (41.3)	40 (40.0)	38 (42.7)	0.77
Type of treatment adjustment, n (%)				
Delay	12 (15.4)	7 (18.0)	5 (13.0)	0.76
Reduce	27 (34.6)	13 (33.0)	14 (37.0)	0.81
Discontinue	23 (29.5)	11 (28.0)	12 (32.0)	0.81
Delay, reduce, and/or discontinue	16 (8.5)	9 (9.0)	7 (7.9)	0.80

^a^The two-item PRO-CTCAE measure evaluates the severity of numbness/tingling in the hands and feet (0 = none, 1 = mild, 2 = moderate, 3 = severe, 4 = very severe) and the degree to which numbness/tingling interfered with usual daily activities (0 = not all, 1 = a little bit, 2 = somewhat, 3 = quite a bit, 4 = very much)

^b^p-value comparing White versus non-White

More than half of the women had been diagnosed with mBC for 1 to 5 years prior (59.8%) though there were significantly more non-White women who had been diagnosed 1 to 5 years prior (p = 0.03). Most women began experiencing CIPN 1–5 years prior (53.0%). Respondents reported a mean score of 1.9 (SD = 0.87) out of 4 for numbness and tingling in the hands and feet, and a mean score of 1.8 (SD = 1.16) out of 4 for interference with daily activities on the PRO-CTCAE sensory neuropathy scale. Non-White women reported a significantly higher mean score for interference with daily activities than White women (p = 0.03). Among the 41.3% of patients whose oncologists recommended treatment adjustments, the majority either underwent dose reductions (34.6%) or discontinued treatment (29.5%).

**Fig. 2:**
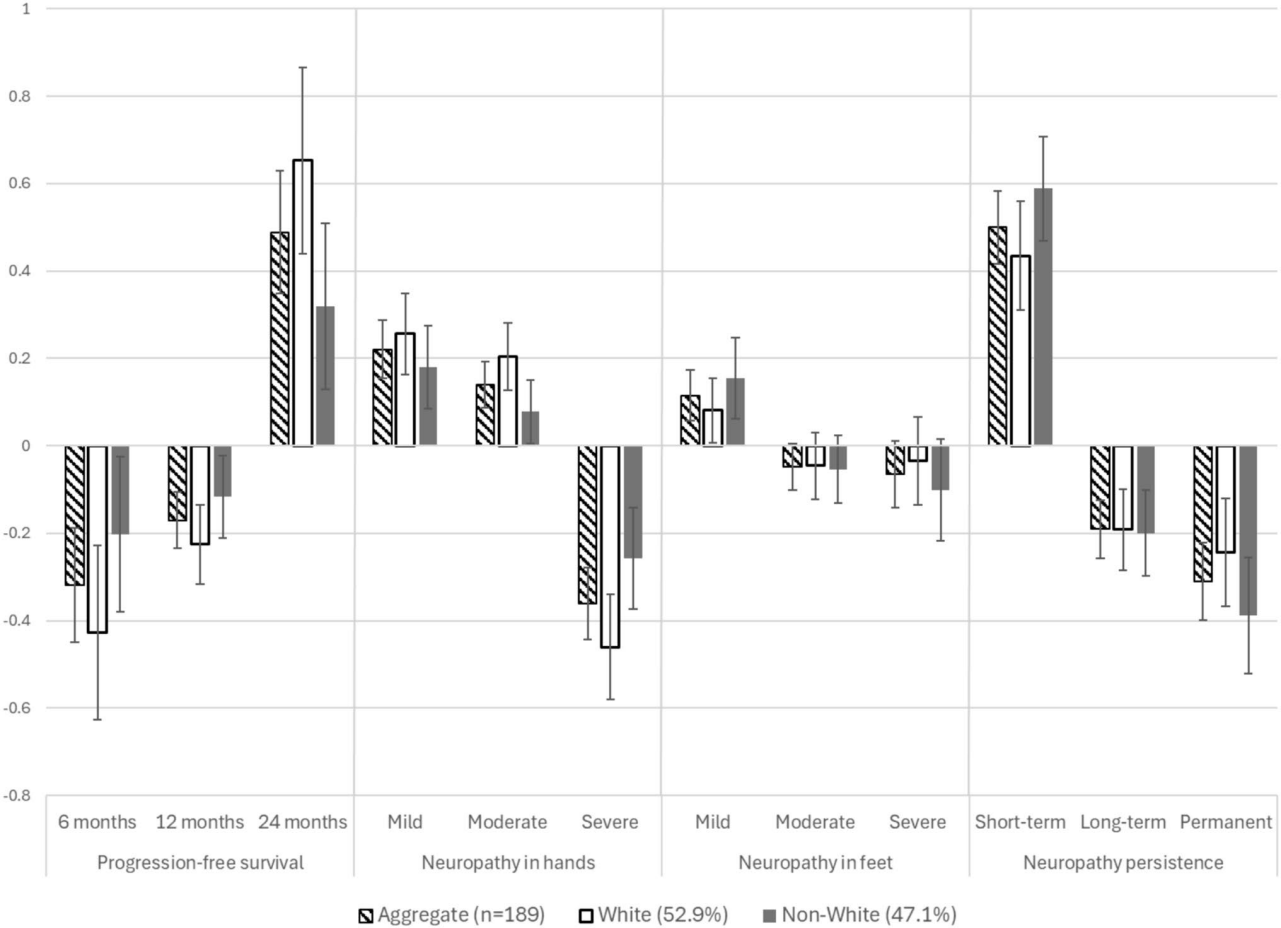
Mean preference weights of attribute levels for both the aggregate and the stratified analysis by race. The vertical bars surrounding each mean preference weight estimate denote the 95% confidence interval around the point estimate. White and non-White women had different preferences (p < 0.01)

Aggregate results of mean preference weights for each attribute’s level show that women favored longer duration of progression-free survival, less severe CIPN in hands and feet, and shorter CIPN duration (Table 3 and Fig. 2).

**Table 3:** Aggregate results of mean preference weights of attribute levels (n = 189)

Choice	*Coefficient	SE	95% CI
Progression-free survival			
24 months	0.49	0.07	0.35, 0.62
12 months	− 0.17	0.03	− 0.23, −0.11
6 months	− 0.32	0.07	− 0.45, −0.19
Neuropathy in hands			
Mild	0.22	0.03	0.15, 0.29
Moderate	0.14	0.03	0.09, 0.19
Severe	− 0.36	0.04	− 0.44, −0.28
Neuropathy in feet			
Mild	0.11	0.03	0.06, 0.17
Moderate	− 0.05	0.03	− 0.10, 0.00
Severe	− 0.07	0.04	− 0.14, 0.01
Neuropathy persistence			
Short-term	0.50	0.04	0.42, 0.58
Long-term	− 0.19	0.03	− 0.26, −0.12
Permanent	− 0.31	0.03	− 0.37, −0.25

^*^Higher mean preference weights (coefficients) suggest greater preference on average for the select attribute level

*SE* standard error

*CI* confidence interval

In the aggregate analysis, respondents tolerated a one-level increase in neuropathy severity in their hands (from mild to moderate, or from moderate to severe) in exchange for 6.7 months of progression-free survival. A one-level increase in neuropathy severity in feet was tolerated in exchange for 2.9 months of progression-free survival. The longest progression-free survival period of 9.3 months was needed for respondents to tolerate a one-level increase in the duration of neuropathy persistence (from short-term to long-term, or from long-term to permanent).

In the stratified analyses by race, similar results were obtained with regard to preferences for longer progression-free survival, lower severity of CIPN in hands and feet, and shorter CIPN duration (Fig. 2). However, non-White women had different preferences from White women (Wald, p < 0.01). Non-White women tolerated a one-level increase in neuropathy severity in their hands in exchange for 8.0 months of progression free survival compared to 6.2 months among White women, and a one-level increase in neuropathy severity in feet in exchange for 5.4 months compared to 1.8 months. Non-white women needed longer progression-free survival benefit to tolerate a one-level increase in the duration of neuropathy persistence (from short-term to long-term, or from long-term to permanent) compared to White women (17.6 versus 5.9 months).

## Discussion

Given the complexity of managing CIPN in clinical practice, measuring patients’ preferences for CIPN risks and survival benefits of continuing neurotoxic chemotherapy has the potential to improve clinician-patient communication and patient-centered outcomes. In this study, women with mBC were willing to tolerate CIPN in exchange for treatment benefits, accepting risks to their hands and feet for fewer months of progression-free survival. However, they were less willing to accept risks associated with persistent CIPN symptoms, requiring more months of progression-free survival to tolerate long-term or permanent effects. Non-White women required more months of progression-free survival benefit to tolerate increases in neuropathy severity of hands or feet and neuropathy duration compared to White women.

In general, women preferred shorter symptom durations and required 9.3 months of progression-free survival before accepting a one-level increase in the duration of neuropathy persistence. Consistent with our findings, Ballinger et al. [31] conducted a choice-based conjoint survey among patients with early-stage breast cancer, presenting taxane treatment choices varying in the degree of benefit, risk of recurrence, and toxicity profile, including CIPN. Patients with CIPN had similar preferences to those who were CIPN-naïve but shifted their preference away from taxanes when CIPN was described as severe or irreversible. Similarly, in a descriptive study, a higher proportion of patients with CIPN (19.1% vs. 6.6%) expressed their willingness to discontinue treatment at moderate CIPN severity if symptoms were to become permanent [32]. These findings highlight the importance of informing patients of the risk of CIPN persistence, especially considering that CIPN symptoms persist in 40–60% of patients three years after treatment [33]. In previous qualitative studies, patients were not aware that CIPN symptoms could be permanent [34–37]. When patients with long-term CIPN were asked what they would have done differently, they stated that they would have stopped treatment early or asked for preventative measures had they known these were options [34]. Without adequate knowledge of CIPN risk and its consequences, patients are not equipped to make informed treatment decisions in collaboration with their oncologists. Unfortunately, it is currently challenging for clinicians to effectively communicate the risk of persistent CIPN to patients due to the absence of predictive tools for persistent CIPN [10]. More data on the incidence and predictors of persistent CIPN is needed to support informed decision-making.

Patients placed a higher value on avoiding neuropathy in their hands than in their feet, as they required more months of progression-free survival to accept a one-level increase in neuropathy severity (6.7 months in hands versus 2.9 months in feet). This study is the first to describe CIPN symptoms in hands and feet as distinct attributes within a choice experiment; thus, comparison with other studies is not possible. Separating attributes for hands and feet is consistent with established CIPN assessment tools, such as the Functional Assessment of Cancer Therapy/Gynecologic Oncology Group-Neurotoxicity (FACT/GOG-Ntx) scale, and the European Organization for Research and Treatment of Cancer (EORTC) Quality of Life (QLQ) Questionnaire-Chemotherapy Induced Peripheral Neuropathy (CIPN20) scale [38, 39]. Two studies found that patients with early-stage breast cancer experience moderate to severe and persistent CIPN symptoms that significantly disrupt their daily life activities post-treatment, particularly in their feet rather than in their hands [40, 41]. Tolerance for risk may vary with increasing CIPN severity, both during and after treatment, and may be particularly influenced by the severity level at the time patients make their decisions [35]. We hypothesize that patients might prioritize avoiding neuropathy in their hands over their feet due to the frequent use of hands in modern life, particularly for activities such as using mobile phones and computers. However, there is no current evidence to support this hypothesis. To better understand the risk tolerance for CIPN in hands versus feet, future research should focus on patient-reported outcomes and quality of life during and after treatment. This information should be complemented with a patient preference survey (such as the one used in this study), where hands and feet are separate attributes, in order to gain deeper insight into the tradeoffs that patients are willing to make.

We found intriguing differences in preferences between White and non-White women. We know from previous literature that incidence of CIPN is greater among self-reported Black patients [15, 16], leading to greater incidence of dose reduction due to CIPN [42] and worse disease-free survival [43]. It is unclear why non-White women were more tolerant of CIPN in the hands but less tolerant of CIPN in the feet than White women. Different types of employment held by each of the groups may be affecting these decisions. A previous study showed that non-Whites had higher rates of work-related physical activity and stronger priorities to maintain function so they could continue to work than Whites [44]. However, there are several other differences between non-White and White patients in our study that could be contributing to these findings. For example, non-White women reported greater functional deficit from CIPN, lower education, lower income, and had been experiencing CIPN for a shorter duration. More in-depth investigation is needed to understand how race and other factors affect treatment preferences and to inform strategies for individualized patient-centered decision-making.

Preference elicitation methods proved invaluable in collecting data on patient choice behavior that would not have been feasible otherwise. This study highlights the average patient perspective when balancing the risk of CIPN progression with the benefits of progression-free survival. Our findings emphasize the need for clinicians to carefully consider the potential permanent effects of CIPN, particularly in patients with moderate to severe CIPN, during the decision-making process. While severe CIPN is less likely to resolve post-treatment [10], communication about the risk of persistent CIPN is still challenging due to several factors, including low numeracy, the lack of predictive tools for CIPN persistence, and the absence of decision aids. In the interim, it is crucial for clinicians to consistently discuss the risks of continuing neurotoxic treatment in relation to CIPN progression, while acknowledging the inherent uncertainties. Beyond monitoring CIPN symptoms, clinicians should guide patients in determining their maximum CIPN tolerance after each treatment cycle [11]. This would involve gauging whether patients could live with their current CIPN severity for the remainder of their lives or if they deemed treatment discontinuation necessary. Equally vital is discussing how these choices might influence treatment effectiveness and exploring alternative treatments if needed. The creation of a decision aid tool tailored for clinical practice remains paramount. Such a tool would facilitate patient-centered care by aligning treatment outcomes with individual patient needs.

This study has some limitations. First, respondents were making decisions independently, without relying on their oncologists. While this approach provided valuable insights from the patient’s perspective, it may not fully mirror real-life choices, where treatment decisions are often made by oncologists or collaboratively between oncologists and patients. In a study by Hertz et al. [32], 61% of patients with CIPN made treatment alteration decisions collaboratively with their clinician. Second, it is possible that patients may not like either of the presented scenarios, but an opt-out option was not included. Excluding an opt-out option allows investigators to better understand tradeoffs between benefits and risks, and is common practice in regulatory benefit-risk analyses [45]. Third, the study data reflects the average preferences of the study participants, and individual patient preferences may differ. Fourth, although multiple security measures were implemented, including personalized survey links and eligibility screening both via email and prior to starting the survey, the information provided by participants was self-reported and could not be independently verified. Finally, health-related decision-making is complex, particularly in the metastatic setting, where patients have undergone multiple treatment regimens and are dealing with competing risks. Further, we did not collect information regarding breast cancer subtypes, which could have provided a more comprehensive description of the sample. The decision to focus solely on CIPN was intentional but may not fully reflect real-life decision-making scenarios in terms of other clinical, financial, or emotional consequences. Additionally, oncologists may recommend alternative non-neurotoxic treatment options to patients that are potentially effective and free from CIPN risk.

## Conclusion

Women with mBC were willing to tolerate CIPN in exchange for treatment benefits, accepting risks to their hands and feet for fewer months of progression-free survival. However, they were less willing to tolerate CIPN risks if symptoms became long-term or permanent. Thus, the duration of symptoms should be carefully discussed and considered when managing CIPN. Significantly different patient preferences were identified between White and non-White women, with non-White women requiring more months of progression-free survival benefit to tolerate increases in neuropathy severity and duration compared to White women. Taking steps to accommodate patients’ preferences when deciding to continue or discontinue treatment due to CIPN may improve treatment experiences and outcomes. Further research is needed to develop a decision aid tool for guiding CIPN-related treatment decisions in clinical practice.

## Supplementary Information

Below is the link to the electronic supplementary material.

Supplementary file1 (DOCX 54 KB)

## Data Availability

The dataset of the current study is available from the corresponding author upon reasonable request.
