# Supplementary material for: Tolerance for chemotherapy-induced peripheral neuropathy among women with metastatic breast cancer: a discrete-choice experiment
Source: Breast Cancer Res Treat. 2025 May 13;212(1):149–59. doi: 10.1007/s10549-025-07715-5 (PMC12086117; doi:10.1007/s10549-025-07715-5)
Supplement: Supplementary file 1 — Supplementary file1 (DOCX 54 KB) [file 10549_2025_7715_MOESM1_ESM.docx]

**Appendix A: Study Survey**

Thank you for your interest in our study. Before we proceed, we need to verify that you are eligible to participate. Please answer the following questions:

Are you 18 years or older?

- Yes
- No

Are you a woman?

- Yes
- No

Do you live in the US?

- Yes
- No

Has a doctor ever told you that you have metastatic breast cancer?

- Yes
- No

What side effects from cancer treatment have you experienced? (Select all that apply)

- Hair loss
- Nausea/vomiting
- Neuropathy (experience of tingling, numbness, muscle weakness, or pain in the hands and feet)
- Neutropenia (a drop in white blood cells count)
- Congestive heart failure (damage to the heart muscle)

**Study title:** Priorities and Preferences for Chemotherapy Discontinuation due to Chemotherapy Induced Peripheral Neuropathy Among Women with Metastatic Breast Cancer

**Purpose of study:** This survey explores your priorities and preferences when discontinuing **chemotherapy treatment (oral or infusion)** because of chemotherapy-induced peripheral neuropathy, or simply neuropathy. Neuropathy is nerve damage that can cause various symptoms in your hands and feet, including pain, numbness, and balance problems. It can make daily tasks difficult and increase your risk of falling. Because there is no cure for neuropathy, your doctor might suggest discontinuing treatment to prevent neuropathy from getting worse.

**Study procedure:** The survey comprises four sections. The first section will ask questions related to your disease, the second section will explore your preferences, and the final section will gather information about you. The survey will take approximately 30 minutes to complete. Because we will ask about neuropathy and its effects on the body, some questions may make you feel uncomfortable. If you wish, you can take a break and come back later using the same device. If you close the survey and reopen it in the same browser, you will be able to continue from where you left off. Please note that you have **1 week** from this day to finish. After that, your response will be recorded as incomplete.

**Voluntary Participation:** This survey is **voluntary** and **anonymous**. You can stop taking the survey at any time. However, to receive compensation for your time ($50), you must complete **all of the questions**.

Do you agree to participate?

- Yes
- No

**Section 1: Medical history and neuropathy**

We will start by asking you some questions about your cancer and the neuropathy symptoms that you have experienced.

When were you diagnosed with metastatic breast cancer?

- Less than 1 year ago
- Between 1 and 5 years ago
- More than 5 years ago

Are you currently receiving treatment for metastatic breast cancer?

- Yes
- No

Which hormonal treatment **have you received or are you still receiving** for **metastatic breast cancer**? (Select all that apply)

- Tamoxifen (Nolvadex)
- Anastrozole (Arimidex)
- Goserelin (Zoldex)
- Letrozole (Femara)
- Leuprorelin (Lupron)
- Exemestane (Aromasin)
- Fulvestrant (Faslodex)
- Elacestrant (Orserdu)
- Triptorelin with Lupron (Trelstar)
- I have not received any hormonal therapy
- Other. Please specify: ____________________

Which treatment regimen are you **currently receiving** for **metastatic breast cancer**? (Select all that apply)

- AC-T (Adriamycin/Doxorbicin, Cyclophosphamide, and Paclitaxel/Taxol)
- AC-T (Adriamycin/Doxorubicin, Cyclophosphamide and Paclitaxel/Taxol with or without Herceptin/Trastuzumab (AC-TH) and Pertuzumab/Perjeta (AC-THP))
- AC-TC-Pembro (Adriamycin/Doxorubicin, Cyclophosphamide, Paclitaxel/Taxol, Carboplatin and Pembrolizumab)
- Affinitor (Everolimus)
- Capecitabine/Xeloda
- Enhertu or Fam-trastuzumab-deruxtecan
- Eribulin, Navelbine, Carboplatin, Cisplatin, Gemcitabine
- Kadcyla or TDM-1
- Neratinib
- Paclitaxel//Taxol or Docetaxel
- TC (Docetaxel and Cyclophosphamide)
- TCH (Taxotere/Docetaxel, Carboplatin, Herceptin/Trastuzumab) with or without Pertuzumab/Perjeta (TCHP)
- THP (Docetaxel or Paclitaxel plus Herceptin/Trastuzumab plus Pertuzumab/Perjeta) Tucatinib/Tukysa plus Capecitabine/Xeloda plus Trastuzumab/Herceptin
- Ibrance (Palbociclib)
- Piqray (Alpelisib)
- Kisqali (Ribociclib)
- Verzenio (Abemaciclib)
- I have not received any of these treatment regimens
- Other. Please specify: ____________________

Which treatment regimen have you **previously received** for **metastatic breast cancer**? (Select all that apply)

- AC-T (Adriamycin/Doxorbicin, Cyclophosphamide, and Paclitaxel/Taxol)
- AC-T (Adriamycin/Doxorubicin, Cyclophosphamide and Paclitaxel/Taxol with or without Herceptin/Trastuzumab (AC-TH) and Pertuzumab/Perjeta (AC-THP))
- AC-TC-Pembro (Adriamycin/Doxorubicin, Cyclophosphamide, Paclitaxel/Taxol, Carboplatin and Pembrolizumab)
- Affinitor (Everolimus)
- Capecitabine/Xeloda
- Enhertu or Fam-trastuzumab-deruxtecan
- Eribulin, Navelbine, Carboplatin, Cisplatin, Gemcitabine
- Everolimus (Affinitor)
- Kadcyla or TDM-1
- Neratinib
- Paclitaxel//Taxol or Docetaxel
- TC (Docetaxel and Cyclophosphamide)
- TCH (Taxotere/Docetaxel, Carboplatin, Herceptin/Trastuzumab) with or without Pertuzumab/Perjeta (TCHP)
- THP (Docetaxel or Paclitaxel plus Herceptin/Trastuzumab plus Pertuzumab/Perjeta)
- Tucatinib/Tukysa plus Capecitabine/Xeloda plus Trastuzumab/Herceptin
- Ibrance (Palbociclib)
- Piqray (Alpelisib)
- Kisqali (Ribociclib)
- Verzenio (Abemaciclib)
- I have not received any of these treatment regimens
- Other. Please specify: ____________________

Are you **currently** experiencing neuropathy (pain, tingling, numbness, or muscle weakness in the hands or feet) from your past or current chemotherapy treatment?

- Yes
- No

How many years has it been since you started experiencing neuropathy from chemotherapy treatment?

- Less than 1 year
- Between 1 and 5 years
- More than 5 years

In the past 7 days, what was the severity of numbness and tingling in your hands or feet at their worst?

- None
- Mild
- Moderate
- Severe
- Very severe

In the past 7 days, how much did numbness or tingling in your hands or feet interfere with your usual or daily activities?

- Not at all
- A little bit
- Somewhat
- Quite a bit
- Very much

Have you ever used medications such as Duloxetine (Cymbalta, Irenka), Pregabalin (Lyrica), Gabapentin (Neurontin), or any other medication to manage neuropathy?

- Yes
- No

Have medications such as Duloxetine (Cymbalta, Irenka), Pregabalin (Lyrica), Gabapentin (Neurontin), or any other medication, helped relieve your neuropathy symptoms?

- Yes
- Not sure
- No

Have you ever used non-drug strategies such as acupuncture, physical therapy, cryotherapy (e.g. frozen gloves and socks), supplements (e.g., vitamin B12), or any other non-drug strategies to manage neuropathy?

- Yes
- No

Have non-drug strategies such as acupuncture, physical therapy, cryotherapy (e.g. frozen gloves and socks), supplements (e.g., vitamin B12), or any other non-drug strategies, helped relieve your neuropathy symptoms?

- Yes
- Not sure
- No

Has your doctor ever recommended altering (reducing, delaying, or discontinuing) chemotherapy treatment due to neuropathy?

- Yes
- No

What changes were performed to your chemotherapy treatment because of neuropathy? (Select all that apply)

- Reduce dose
- Delay treatment
- Discontinue treatment and switch to another therapy

Do you think that chemotherapy treatment alteration improved your neuropathy symptoms?

- Yes
- No

Do you have any regrets about altering chemotherapy treatment because of neuropathy?

- Yes
- No

**Section 2: Identifying the maximum acceptable neuropathy risk that patients are willing to tolerate in exchange for the benefit of progression-free survival**

Chemotherapy treatment that causes neuropathy has both risks and benefits. We will show you a number of risks and benefits with varying levels. These include progression-free survival (PFS), neuropathy in hands, neuropathy in feet, and neuropathy persistence.

We want to know how important the following risks and benefits are to you when deciding to continue or discontinue treatment because of neuropathy. Note that these are hypothetical risk and benefit levels that may not be relevant to your personal experience.

**Progression-free survival (PFS)**

The length of time that the treatment is effective in controlling the growth of cancer before the cancer starts to grow again. The different levels of progression-free survival (PFS) include:

6 months of PFS

12 months of PFS

24 months of PFS

**How important is improving your PFS?**

- Not important
- Important
- Very important

**Neuropathy in hands**

Chemotherapy treatment can cause tingling, numbness, burning or sharp pain, and muscle weakness starting from the fingers and spreading to the hands, wrists, and even elbows. Symptoms may affect your ability to perform activities of daily living, such as doing or undoing buttons or zips, using a telephone, or holding a cup. The different levels of neuropathy in the hands include:

| Mild | Symptoms do not affect activities of daily living |
| --- | --- |
| Moderate | Symptoms affect activities of daily living but do not completely prevent individuals from doing these activities |
| Severe | Symptoms significantly affect activities of daily living, to the point where individuals may require assistance |

**How important is reducing the risk of neuropathy in your hands?**

- Not important
- Important
- Very important

**Neuropathy in feet**

Chemotherapy treatment can cause tingling, numbness, burning or sharp pain, and muscle weakness starting from toes and spreading to the feet, ankles, and even knees. Symptoms may affect your ability to perform activities of daily living such as walking, climbing stairs, and running. Symptoms can also increase your risk of falling because of losing balance. The different levels of neuropathy in the feet include:

| Mild | Symptoms do not affect activities of daily living, nor do they increase your risk of falling |
| --- | --- |
| Moderate | Symptoms affect activities of daily living but do not increase your risk of falling Severe Symptoms affect both activities of daily living and increase your risk of falling. |
| Severe | Symptoms affect both activities of daily living and increase your risk of falling. Individuals may require physical therapy or the use of mobility aids such as crutches, walkers, or a wheelchair |

**How important is reducing the risk of neuropathy in your feet?**

- Not important
- Important
- Very important

**Neuropathy persistence**

The length of time that your neuropathy symptoms continue even after discontinuing the course of treatment that was causing your neuropathy. The different levels of neuropathy persistence include:

Short-term neuropathy will last 6 months after treatment discontinuation

Long-term neuropathy will last 12 months after treatment discontinuation

Permanent neuropathy will last forever after treatment discontinuation

**How important is reducing the risk of neuropathy persistence?**

- Not important
- Important
- Very important

Now, consider this other situation: Sara’s doctor tells her that continuing chemotherapy treatment may increase the risk of neuropathy severity and persistence but may improve the benefit of progression-free survival (PFS).

If you were Sara, how much neuropathy severity and persistence are you willing to tolerate in exchange for the benefit of progression-free survival (PFS) before deciding to discontinue chemotherapy treatment because of neuropathy?

First, we will show you an example of what Sara chose, then in the next page, it will be your turn to choose.

**Here is the example:**

Sara was shown the following scenarios with varying levels of the risks and benefits described before, and asked to select the scenario where she would be willing to tolerate neuropathy severity in exchange for the benefit of progression-free survival (PFS).

| Attributes | Scenario A | Scenario B |
| --- | --- | --- |
| Progression-free survival | 12 months | 6 months |
| Neuropathy in hands | Severe | Moderate |
| Neuropathy in feet | Severe | Moderate |
| Neuropathy persistence | Permanent | Long-term |
| Under which scenario would you continue treatment? | **☐** | **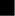** |

Sara chose scenario B over scenario A because she preferred experiencing temporary and moderate neuropathy in her hands and feet, over longer progression-free survival (PFS) accompanied by severe and permanent neuropathy in scenario A.

**Now, it's your turn:**

In the next pages, we will show you 12 tables similar to the one you saw before. Each table includes two scenarios, each with different levels of risks and benefits. For each table, choose the scenario where you would be willing to **continue** treatment after weighing the risks of neuropathy severity and persistence against the benefit of progression-free survival (PFS). Select either scenario A or scenario B. Note that these are hypothetical scenarios that may not be relevant to your personal experience.

For a reminder of the risks and benefits and respective levels, download this [Table](https://vcupharmacy.co1.qualtrics.com/CP/File.php?F=F_8GQPXlkh3IZvav4).

| Attributes | Scenario A | Scenario B |
| --- | --- | --- |
| Progression-free survival | 12 months | 24 months |
| Neuropathy in hands | Moderate | Severe |
| Neuropathy in feet | Mild | Severe |
| Neuropathy persistence | Permanent | Long-term |
| Under which scenario would you continue treatment? | **☐** | **☐** |

| Attributes | Scenario A | Scenario B |
| --- | --- | --- |
| Progression-free survival | 24 months | 12 months |
| Neuropathy in hands | Severe | Mild |
| Neuropathy in feet | Mild | Severe |
| Neuropathy persistence | Permanent | Short-term |
| Under which scenario would you continue treatment? | **☐** | **☐** |

| Attributes | Scenario A | Scenario B |
| --- | --- | --- |
| Progression-free survival | 12 months | 6 months |
| Neuropathy in hands | Severe | Moderate |
| Neuropathy in feet | Severe | Moderate |
| Neuropathy persistence | Permanent | Long-term |
| Under which scenario would you continue treatment? | **☐** | **☐** |

| Attributes | Scenario A | Scenario B |
| --- | --- | --- |
| Progression-free survival | 24 months | 6 months |
| Neuropathy in hands | Mild | Moderate |
| Neuropathy in feet | Mild | Moderate |
| Neuropathy persistence | Short-term | Permanent |
| Under which scenario would you continue treatment? | **☐** | **☐** |

| Attributes | Scenario A | Scenario B |
| --- | --- | --- |
| Progression-free survival | 12 months | 6 months |
| Neuropathy in hands | Moderate | Severe |
| Neuropathy in feet | Severe | Mild |
| Neuropathy persistence | Long-term | Short-term |
| Under which scenario would you continue treatment? | **☐** | **☐** |

| Attributes | Scenario A | Scenario B |
| --- | --- | --- |
| Progression-free survival | 24 months | 12 months |
| Neuropathy in hands | Mild | Severe |
| Neuropathy in feet | Moderate | Severe |
| Neuropathy persistence | Long-term | Short-term |
| Under which scenario would you continue treatment? | **☐** | **☐** |

| Attributes | Scenario A | Scenario B |
| --- | --- | --- |
| Progression-free survival | 6 months | 24 months |
| Neuropathy in hands | Moderate | Mild |
| Neuropathy in feet | Mild | Moderate |
| Neuropathy persistence | Short-term | Permanent |
| Under which scenario would you continue treatment? | **☐** | **☐** |

| Attributes | Scenario A | Scenario B |
| --- | --- | --- |
| Progression-free survival | 6 months | 12 months |
| Neuropathy in hands | Mild | Severe |
| Neuropathy in feet | Severe | Moderate |
| Neuropathy persistence | Permanent | Permanent |
| Under which scenario would you continue treatment? | **☐** | **☐** |

| Attributes | Scenario A | Scenario B |
| --- | --- | --- |
| Progression-free survival | 24 months | 6 months |
| Neuropathy in hands | Moderate | Mild |
| Neuropathy in feet | Moderate | Mild |
| Neuropathy persistence | Short-term | Long-term |
| Under which scenario would you continue treatment? | **☐** | **☐** |

| Attributes | Scenario A | Scenario B |
| --- | --- | --- |
| Progression-free survival | 6 months | 12 months |
| Neuropathy in hands | Severe | Mild |
| Neuropathy in feet | Severe | Mild |
| Neuropathy persistence | Long-term | Permanent |
| Under which scenario would you continue treatment? | **☐** | **☐** |

| Attributes | Scenario A | Scenario B |
| --- | --- | --- |
| Progression-free survival | 6 months | 24 months |
| Neuropathy in hands | Mild | Moderate |
| Neuropathy in feet | Moderate | Mild |
| Neuropathy persistence | Short-term | Long-term |
| Under which scenario would you continue treatment? | **☐** | **☐** |

| Attributes | Scenario A | Scenario B |
| --- | --- | --- |
| Progression-free survival | 12 months | 24 months |
| Neuropathy in hands | Severe | Moderate |
| Neuropathy in feet | Moderate | Severe |
| Neuropathy persistence | Long-term | Short-term |
| Under which scenario would you continue treatment? | **☐** | **☐** |

**Section 3: Demographic and psychosocial characteristics**

Lastly, please answer the following questions about yourself.

What is your age? [Text-box]

What is your marital status?

- Single
- In a relationship

Do you have children who are younger than 18 years old?

- Yes
- No

Which of the following racial or ethnic groups best describes you?

- Black, non-Hispanic
- White, non-Hispanic
- Hispanic
- Other, non-Hispanic

What is the highest level of education you have completed?

- High school or less
- Some college
- Bachelor’s degree or higher

What state do you live in? [drop-down menu]

What is your household income?

- Less than 34,999
- 35,000–84,999
- More than 85,000
- Prefer not to say

These questions ask you about your ability to understand cancer-related medical information.

The normal range for hemoglobin for a male is 13.3 - 17.2 g/dL. Joe’s hemoglobin is 9.7 g/dL. Is Joe within the normal range?

- Yes
- No

A biopsy of a tumor is done to...

- Remove it
- Diagnose it
- Treat it

If a patient has stage 1 cancer, it means the cancer is...

- Localized
- In nearby organ
- In distant sites

The role of a physical therapist is to talk to a patient about emotional needs

- True
- False

A tumor is considered ‘‘inoperable’’ when it cannot be treated with...

- Radiation therapy
- Surgery
- Chemotherapy

Sally will get radiation therapy once a day, Monday through Friday. If Sally has therapy for 4 weeks, how many times will she get radiation therapy?

- 5
- 15
- 20

These questions ask about your relationship with the doctor who prescribed your chemotherapy. Indicate the extent you agree or disagree with the following statements.

| Statement | Strongly  disagree | Disagree | As much agree as  disagree | Agree | Strongly  agree |
| --- | --- | --- | --- | --- | --- |
| Your doctor is totally honest in telling you about all the different treatment options available for your condition | O | O | O | O | O |
| You think your doctor can handle any medical situation, even a very serious one | O | O | O | O | O |
| Your doctor listens with care and concern to all the problems you have | O | O | O | O | O |
| Your doctor will do whatever it takes to get you all the care you need | O | O | O | O | O |
| All in all, you have complete trust in your doctor | O | O | O | O | O |

| I worry about…. | Not at all | A little bit | Somewhat | Very much |
| --- | --- | --- | --- | --- |
| Future diagnostic tests | O | O | O | O |
| Another type of cancer | O | O | O | O |
| Dying | O | O | O | O |
| My health | O | O | O | O |
| My children’s health | O | O | O | O |

These questions ask you to indicate the degree to which you have fears about cancer recurrence and health using the 1 (not at all) to 4 (very much) scale.
